# Pregnancy, Fertility, Breastfeeding, and Alcohol Consumption: An Analysis of Framing and Completeness of Information Disseminated by Alcohol Industry–Funded Organizations

**DOI:** 10.15288/jsad.2019.80.524

**Published:** 2019-10-13

**Authors:** Audrey W. Y. Lim, May C. I. Van Schalkwyk, Nason Maani Hessari, Mark P. Petticrew

**Affiliations:** ^a^ Faculty of Public Health and Policy, London School of Hygiene and Tropical Medicine, London, United Kingdom; ^b^ Public Health Policy Evaluation Unit, School of Public Health, Imperial College London, London, United Kingdom

## Abstract

**Objective:**

Alcohol use during pregnancy can harm the developing fetus. The exact amount, pattern, and critical period of exposure necessary for harm to occur are unclear, although official guidance often emphasizes precautionary abstention. The impacts on fertility and breastfeeding are also unclear. Information on alcohol and pregnancy is disseminated by the alcohol industry–funded organizations, and there are emerging concerns about its accuracy, suggesting the need for detailed analysis.

**Method:**

Information on alcohol consumption in relation to fertility, pregnancy, and breastfeeding was extracted from the websites of 23 alcohol industry–funded bodies (e.g., Drinkaware [United Kingdom] and DrinkWise [Australia]), and 19 public health organizations (e.g., Health.gov and NHS Choices). Comparative qualitative and quantitative analysis of the framing and completeness of this information was undertaken.

**Results:**

Alcohol industry–funded organizations were statistically significantly less likely than public health websites to provide information on fetal alcohol spectrum disorder and less likely to advise that no amount of alcohol is safe during pregnancy. They were significantly more likely to emphasize uncertainties and less likely to use direct language (e.g., “don’t drink”). Some alcohol industry–funded (and no public health) websites appear to use “alternate causation” arguments, similar to those used by the tobacco industry, to argue for causes of alcohol harms in pregnancy other than alcohol.

**Conclusions:**

Alcohol industry–funded websites omit and misrepresent the evidence on key risks of alcohol consumption during pregnancy. This may “nudge” women toward continuing to drink during pregnancy. These findings suggest that alcohol industry–funded bodies may increase risk to pregnant women by disseminating misinformation. The public should be made widely aware of the risks of obtaining health information from alcohol industry–funded sources.

Alcohol is a teratogen that, when consumed during pregnancy, can cross the placenta and damage the brain and other organs of the developing embryo and fetus (Caputo et al., 2016). The risks include fetal alcohol spectrum disorder (FASD), which refers to the range of adverse health effects of prenatal exposure to alcohol, including brain damage, congenital anomalies, and cognitive, behavioral, emotional, and adaptive functioning deficits (O’Keeffe et al., 2014; Ruisch et al., 2018). One in every 13 pregnant women who consume alcohol during pregnancy is estimated to have a child with FASD, although there are difficulties in obtaining a reliable and valid diagnosis (Lange et al., 2017).

Lange et al. (2017) note that research on humans has not been able to determine the pattern, amount, and/or critical period of prenatal alcohol exposure necessary for harm to the developing fetus, although animal models show harm at all stages of embryonic development (Lange et al., 2017). Evidence of the harmful effects of drinking less than 32 g of alcohol per week in pregnancy is limited, but even light prenatal alcohol consumption is associated with being small for gestational age and with preterm delivery (Mamluk et al., 2017). The U.K. guidelines on alcohol consumption in pregnancy advise, “If you are pregnant or planning a pregnancy, the safest approach is not to drink alcohol at all, to keep risks to your baby to a minimum” (Alcohol Policy Team, Department of Health, 2016), and other health guidelines take a similar precautionary approach (Butt et al., 2011; National Health and Medical Research Council, 2009).

There is limited research on the impact on male and female fertility, although alcohol has been shown to have negative effects on male reproductive systems (Salas-Huetos et al., 2017). There is little evidence for harms from occasional alcohol intake (Ricci et al., 2017). A recent systematic review concluded that female alcohol consumption was associated with a 13% reduction in the likelihood of pregnancy (Fan et al., 2017). There is limited research on the effect of alcohol consumption during breastfeeding on infant development, although adverse effects include early cessation of breastfeeding, disruption of infant-feeding behavior, and sleeping-time reduction (Wilson et al., 2017). A recent review concluded that the effects during lactation remain unknown and advised following standard recommendations on alcohol consumption (Haastrup et al., 2014).

There are many public sources of information on these issues. Alongside government-sponsored health websites, the alcohol industry also disseminates information via corporate social responsibility organizations (e.g., Drinkaware [United Kingdom] and DrinkWise [Australia]). Corporate social responsibility activities, which include education campaigns on the harms of their products (Babor & Robaina, 2013; Gilmore et al., 2011; McCambridge et al., 2018), help companies reduce commercial risks. Such organizations selectively frame alcohol-related harms in terms of the individual consumer and “responsible consumption,” rather than in terms of harmful products (Mialon & McCambridge, 2018). These organizations have also been shown to disseminate misleading information on cancer and alcohol consumption, for example, by emphasizing the potential confounding effects of non-alcohol risk factors (Petticrew et al., 2018a, 2018b).

Posters on alcohol and pregnancy produced by the Australian alcohol industry–funded organization DrinkWise have been withdrawn for providing inaccurate information (Han, 2018). Their revised text has also been claimed to use confusing, indirect language—for example, by not actively advising abstention but by framing it as a choice and avoiding stating that drinking during pregnancy can cause harm (Koplin, 2018). It is within this context that we aimed to analyze the information on pregnancy, fertility, and breastfeeding provided by international alcohol industry corporate social responsibility bodies to assess the framing and completeness of the information provided to the public.

## Method

The study systematically analyzed the completeness and framing of the information on reproductive health topics (fertility, pregnancy, breastfeeding, and fetal health) provided by international alcohol industry corporate social responsibility organizations when compared with information provided by national public health information websites from a sample of English-speaking countries. Alcohol industry–funded organizations were identified by searching the Global Alcohol Producers website and its progress reports, and from the corporate social responsibility sections of alcohol producers’ websites. This identification was checked against a previously published list (Petticrew et al., 2018a). We excluded inaccessible websites and those containing only corporate information. The websites of 23 organizations in total were available for analysis (Table 1). Searches were conducted September 4, 2018, and updated in January 2019.

**Table 1.**
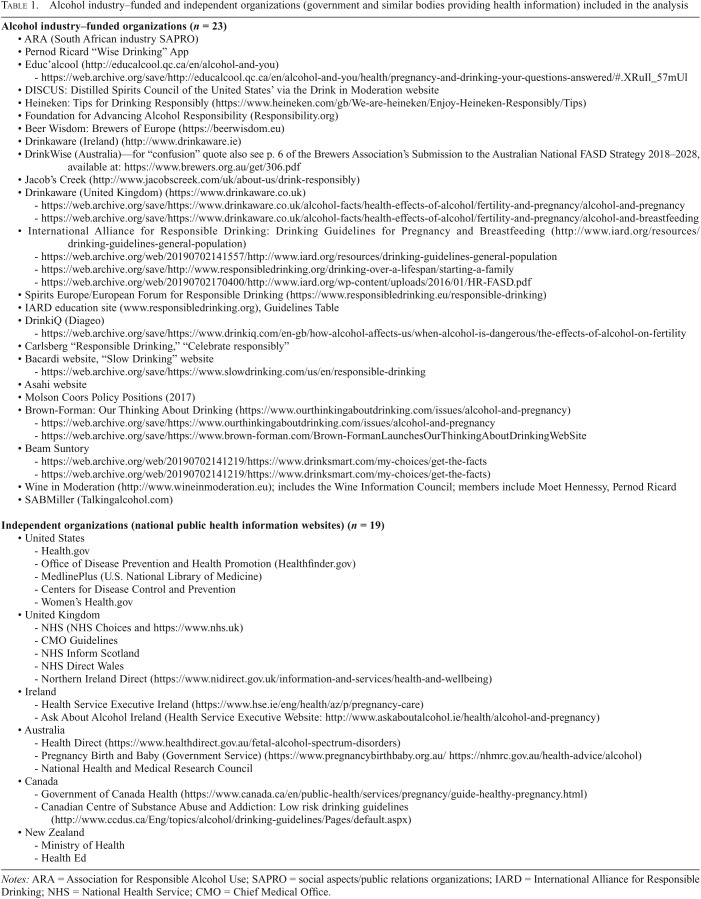
Alcohol industry–funded and independent organizations (government and similar bodies providing health information) included in the analysis

**Alcohol industry–funded organizations (*n* = 23)**	
• ARA (South African industry SAPRO)	
• Pernod Ricard “Wise Drinking” App	
• Educ’alcool (http://educalcool.qc.ca/en/alcohol-and-you)	
- https://web.archive.org/save/http://educalcool.qc.ca/en/alcohol-and-you/health/pregnancy-and-drinking-your-questions-answered/#.XRuIl_57mUl	
• DISCUS: Distilled Spirits Council of the United States’ via the Drink in Moderation website	
• Heineken: Tips for Drinking Responsibly (https://www.heineken.com/gb/We-are-heineken/Enjoy-Heineken-Responsibly/Tips)	
• Foundation for Advancing Alcohol Responsibility (Responsibility.org)	
• Beer Wisdom: Brewers of Europe (https://beerwisdom.eu)	
• Drinkaware (Ireland) (http://www.drinkaware.ie)	
• DrinkWise (Australia)—for “confusion” quote also see p. 6 of the Brewers Association’s Submission to the Australian National FASD Strategy 2018–2028, available at: https://www.brewers.org.au/get/306.pdf	
• Jacob’s Creek (http://www.jacobscreek.com/uk/about-us/drink-responsibly)	
• Drinkaware (United Kingdom) (https://www.drinkaware.co.uk)	
- https://web.archive.org/save/https://www.drinkaware.co.uk/alcohol-facts/health-effects-of-alcohol/fertility-and-pregnancy/alcohol-and-pregnancy	
- https://web.archive.org/save/https://www.drinkaware.co.uk/alcohol-facts/health-effects-of-alcohol/fertility-and-pregnancy/alcohol-and-breastfeeding
• International Alliance for Responsible Drinking: Drinking Guidelines for Pregnancy and Breastfeeding (http://www.iard.org/resources/drinking-guidelines-general-population)
- https://web.archive.org/web/20190702141557/http://www.iard.org/resources/drinking-guidelines-general-population
- https://web.archive.org/save/http://www.responsibledrinking.org/drinking-over-a-lifespan/starting-a-family
- https://web.archive.org/web/20190702170400/http://www.iard.org/wp-content/uploads/2016/01/HR-FASD.pdf
• Spirits Europe/European Forum for Responsible Drinking (https://www.responsibledrinking.eu/responsible-drinking)
• IARD education site (www.responsibledrinking.org), Guidelines Table
• DrinkiQ (Diageo)
- https://web.archive.org/save/https://www.drinkiq.com/en-gb/how-alcohol-affects-us/when-alcohol-is-dangerous/the-effects-of-alcohol-on-fertility
• Carlsberg “Responsible Drinking,” “Celebrate responsibly”
• Bacardi website, “Slow Drinking” website
- https://web.archive.org/save/https://www.slowdrinking.com/us/en/responsible-drinking
• Asahi website
• Molson Coors Policy Positions (2017)
• Brown-Forman: Our Thinking About Drinking (https://www.ourthinkingaboutdrinking.com/issues/alcohol-and-pregnancy)
- https://web.archive.org/save/https://www.ourthinkingaboutdrinking.com/issues/alcohol-and-pregnancy
- https://web.archive.org/save/https://www.brown-forman.com/Brown-FormanLaunchesOurThinkingAboutDrinkingWebSite
• Beam Suntory
- https://web.archive.org/web/20190702141219/https://www.drinksmart.com/my-choices/get-the-facts
- https://web.archive.org/web/20190702141219/https://www.drinksmart.com/my-choices/get-the-facts)
• Wine in Moderation (http://www.wineinmoderation.eu); includes the Wine Information Council; members include Moet Hennessy, Pernod Ricard
• SABMiller (Talkingalcohol.com)
**Independent organizations (national public health information websites) (*n* = 19)**
• United States
- Health.gov
- Office of Disease Prevention and Health Promotion (Healthfinder.gov)
- MedlinePlus (U.S. National Library of Medicine)
- Centers for Disease Control and Prevention
- Women’s Health.gov
• United Kingdom
- NHS (NHS Choices and https://www.nhs.uk)
- CMO Guidelines
- NHS Inform Scotland
- NHS Direct Wales
- Northern Ireland Direct (https://www.nidirect.gov.uk/information-and-services/health-and-wellbeing)
• Ireland
- Health Service Executive Ireland (https://www.hse.ie/eng/health/az/p/pregnancy-care)
- Ask About Alcohol Ireland (Health Service Executive Website: http://www.askaboutalcohol.ie/health/alcohol-and-pregnancy)
• Australia
- Health Direct (https://www.healthdirect.gov.au/fetal-alcohol-spectrum-disorders)
- Pregnancy Birth and Baby (Government Service) (https://www.pregnancybirthbaby.org.au/ https://nhmrc.gov.au/health-advice/alcohol)
- National Health and Medical Research Council
• Canada
- Government of Canada Health (https://www.canada.ca/en/public-health/services/pregnancy/guide-healthy-pregnancy.html)
- Canadian Centre of Substance Abuse and Addiction: Low risk drinking guidelines
(http://www.ccdus.ca/Eng/topics/alcohol/drinking-guidelines/Pages/default.aspx)
• New Zealand
- Ministry of Health
- Health Ed

*Notes:* ARA = Association for Responsible Alcohol Use; SAPRO = social aspects/public relations organizations; IARD = International Alliance for Responsible Drinking; NHS = National Health Service; CMO = Chief Medical Office.

For comparison, we searched the health websites of government bodies from the United States, United Kingdom, Canada, Australia, Ireland, and New Zealand (*n* = 19). Websites were included if they were national government-sponsored resources providing health information. Although there are websites that specifically aim to provide information on pregnancy (e.g., the websites of charities focused solely on fetal alcohol syndrome [FAS]), we did not consider these to be a similar enough comparator. We expected that these websites would differ significantly from the alcohol industry–funded websites in the amount of detailed information they provide. Such a comparison would therefore be biased against the industry–funded websites, because corporate social responsibility websites, similar to government health websites, are not solely focused on pregnancy but include a range of other health information.

All text on the relevant health issue was extracted into Excel tables by three researchers working independently, with differences resolved by jointly checking the original data source. Two researchers iteratively coded all the data, through which we identified 10 themes (Table 2), from which the 4 main qualitative themes were identified. Three coders then independently checked all extracted text against the codes. We assessed coder reliability by calculating a kappa statistic for multiple raters across the 10 themes in Table 3. Mean Fleiss’ κ was .74, indicating substantial agreement (Fleiss, 1971).

**Table 2.**
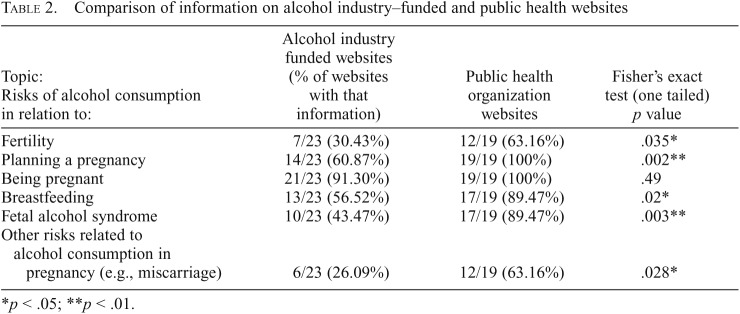
Comparison of information on alcohol industry–funded and public health websites

Topic: Risks of alcohol consumption in relation to:	Alcohol industry funded websites (% of websites with that information)	Public health organization websites	Fisher’s exact test (one tailed) *p* value
Fertility	7/23 (30.43%)	12/19 (63.16%)	.035*
Planning a pregnancy	14/23 (60.87%)	19/19 (100%)	.002**
Being pregnant	21/23 (91.30%)	19/19 (100%)	.49
Breastfeeding	13/23 (56.52%)	17/19 (89.47%)	.02*
Fetal alcohol syndrome	10/23 (43.47%)	17/19 (89.47%)	.003**
Other risks related to alcohol consumption in pregnancy (e.g., miscarriage)	6/23 (26.09%)	12/19 (63.16%)	.028*

* *p* < .05; **p < .01.

**Table 3.**
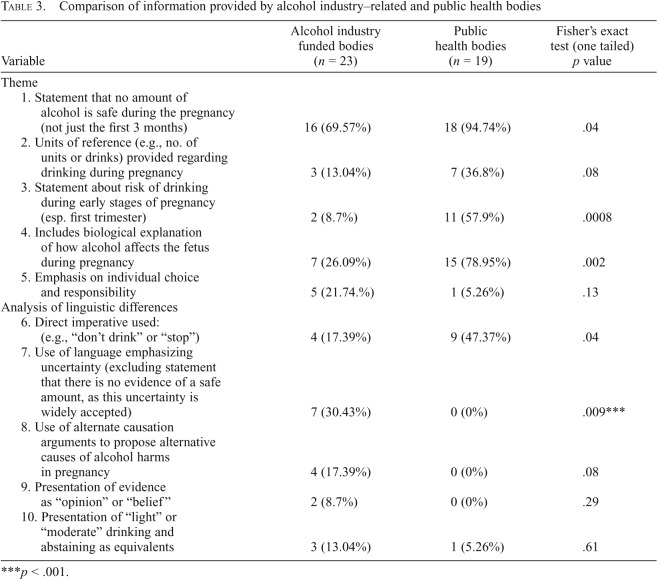
Comparison of information provided by alcohol industry–related and public health bodies

Variable	Alcohol industry funded bodies (*n* = 23)	Public health bodies (*n* = 19)	Fisher’s exact test (one tailed) *p* value
Theme			
1. Statement that no amount of alcohol is safe during the pregnancy (not just the first 3 months)	16 (69.57%)	18 (94.74%)	.04
2. Units of reference (e.g., no. of units or drinks) provided regarding drinking during pregnancy	3 (13.04%)	7 (36.8%)	.08
3. Statement about risk of drinking during early stages of pregnancy (esp. first trimester)	2 (8.7%)	11 (57.9%)	.0008
4. Includes biological explanation of how alcohol affects the fetus during pregnancy	7 (26.09%)	15 (78.95%)	.002
5. Emphasis on individual choice and responsibility	5 (21.74.%)	1 (5.26%)	.13
Analysis of linguistic differences			
6. Direct imperative used: (e.g., “don’t drink” or “stop”)	4 (17.39%)	9 (47.37%)	.04
7. Use of language emphasizing uncertainty (excluding statement that there is no evidence of a safe amount, as this uncertainty is widely accepted)	7 (30.43%)	0 (0%)	.009***
8. Use of alternate causation arguments to propose alternative causes of alcohol harms in pregnancy	4 (17.39%)	0 (0%)	.08
9. Presentation of evidence as “opinion” or “belief”	2 (8.7%)	0 (0%)	.29
10. Presentation of “light” or “moderate” drinking and abstaining as equivalents	3 (13.04%)	1 (5.26%)	.61

*** *p* < .001.

### Analysis methods

#### Gap analysis of provision of information on alcohol industry–funded websites compared with public health websites.

To identify omissions, alcohol industry–funded websites and public health websites were coded if they provided information on alcohol consumption plus any information on fertility, planning a pregnancy, pregnancy, breastfeeding, FASD, or “other risks including miscarriage.”

#### Thematic analysis.

Open coding of the data identified 10 themes; from these, 4 substantive themes for further qualitative analysis were identified. We coded only content on the organization’s webpages; additional content was not coded if it derived from a separate organization and so would not necessarily represent the “voice” of the organization.

#### Quantitative analysis comparing alcohol industry–funded and non–alcohol industry websites.

We compared the frequency of the appearance of different types of information on the two types of websites using Fisher’s exact test, in the Centers for Disease Control and Prevention’s Epi Info package (Version 7.2) (Table 2). We also analyzed the language used, hypothesizing that language on industry-funded websites would be less likely to use direct speech (e.g., “don’t drink”). This is a characteristic of alcohol industry arguments (Koplin, 2018). Given previous findings (Koplin, 2018), we also examined the use of language emphasizing uncertainties, the use of alternative causation arguments (e.g., proposing alternative, non-alcohol causes of harms in pregnancy), the presentation of evidence as a matter of opinion or belief, and the presentation of “light” or “moderate” drinking as being equivalent to abstaining.

## Results

### Gap analysis: Alcohol industry–funded websites compared with public health websites

There was no significant difference between alcohol industry–funded bodies and public health bodies in the likelihood of including general information on alcohol and pregnancy (Table 2); however, there are many significant differences in terms of the specific information that is presented, and how it is presented. In particular, the health sections of the websites of alcohol industry–funded organizations were significantly less likely than those of public health organizations to include information on most topics relevant to fertility, pregnancy, breastfeeding, and FAS/FASD. In the case of FAS, fewer than half of alcohol industry–funded organizations included this information, compared with approximately 90% of public health organizations.

The websites of alcohol industry–related bodies were also significantly less likely to include information on most pregnancy-related harms. Some websites include a range of other health information but omit information on pregnancy. For example, the Beam Suntory “Drink Smart” website “How Alcohol Affects You” section contains a large amount of nonspecific advice, such as “excessive drinking leads to dangerous health effects,” but does not mention pregnancy or FAS/FASD.

### Thematic analysis: Analysis of information provided by alcohol industry–funded bodies

It has been found previously that alcohol industry–funded bodies highlight uncertainties in the evidence on health harms and frame those harms in such a way as to deflect responsibility from the industry itself. We identified four such approaches (i.e., themes), as follows.

#### Emphasizing uncertainty and implying safety.

Several alcohol industry–funded websites appear to emphasize the scientific uncertainties regarding safe levels of drinking. The International Alliance for Responsible Drinking (IARD), an alcohol producers’ “responsible drinking” body, appears to emphasize uncertainty regarding “safe” limits by publishing a table that details “drinking guidelines for alcohol consumption by women who may become pregnant, are pregnant and are breastfeeding issued by government bodies in various countries.” The table shows national guidelines from Albania to Vietnam, with no accompanying explanation. The variation is arguably obvious to the reader, with visible gaps where no pre-pregnancy or breastfeeding guidelines are provided. IARD is the successor organization to the International Center for Alcohol Policies. An analysis of IARD’s purpose and activities has found that much of these guidelines focused on countering the influence of both the World Health Organization and leading alcohol researchers (Jernigan, 2012). Lack of consensus is also highlighted by Brown-Forman, which references the “ongoing debate about whether there is a ‘safe’ level of consumption during pregnancy, or during certain time frames of a woman’s pregnancy.” The word *debate* is commonly used elsewhere in alcohol and tobacco industry narratives to imply that scientific evidence is simply a matter of debate or opinion among scientists (Babor, 2009; Babor & Robaina, 2013; Brandt, 2012). DrinkWise also states that there is “confusion about how much one can safely drink during pregnancy”—with the added apparent implication that such a safe level exists.

Drinkaware appears to take a similar approach, apparently promoting lack of trust in pregnancy information in general: “Understandably it can be hard to know where to go for trustworthy advice. This is especially true when it comes to advice about drinking alcohol when you’re pregnant.”

Some wording appears to imply that alcohol is safe—but has not yet been proven to be so. For example, Diageo’s DrinkiQ website states that “research has *yet* [emphasis added] to establish a ‘safe’ amount to drink during pregnancy,” whereas Educ’alcool Canada (an alcohol industry–funded organization involving alcoholic beverage industry associations and Quebec public health organizations) highlights the gaps in the evidence: “*To date,* [emphasis added] researchers have not been able to determine the exact amount of alcohol that is completely safe for the development of the fetus, even though there is no evidence that the occasional drink has any harmful effect.” The language in the first clause of this sentence implies that there is a completely safe limit, which simply has not yet been identified. Coupled with the second half of the sentence “. . . there is no evidence that the occasional drink has any harmful effect,” the advice appears to endorse drinking in pregnancy. The website advice continues: “We do know, however, that the risk of miscarriage, birth defects, growth retardation and mental disorders increases the more drinks the mother has on each occasion, and the more frequently she drinks. The scientific community *believes* [emphasis added] that abstaining from drinking is the safest choice.” This appears to be an example of industry “mixed messages.” The word *believe* (like the word *debate*) may also imply that that this message is based not on evidence but on ideology.

#### Framing information to emphasize individual responsibility, drinking patterns, and individual variation and choice.

Several alcohol industry–funded bodies introduce the idea of risk associated with different patterns of drinking, suggesting that some patterns are more harmful than others at different stages of the reproductive process. Industry “responsible drinking” campaigns often do this by encouraging people to “know their limits” individually and “choose what is right for them” (Maani Hessari & Petticrew, 2018). For example, although Bacardi does identify drinking during pregnancy as “risky,” it prefaces this with the claim that “what is ‘too much’ may vary by individual.” Ambiguity may also be seen in Canada Educ’alcool’s statement that “[t]he risk to the fetus is reduced considerably if you have only one drink every now and then.”

Individualization of risk, choice, and ambiguity, is also seen in Drinkaware’s information on pregnancy and breastfeeding. Within a section titled “Choosing what is right for you,” Drinkaware quotes a Royal College of Midwives advisor who states that “The RCM advises abstinence in pregnancy and during breastfeeding . . . in light of all the evidence, we believe cumulative alcohol consumption can be harmful to mother and baby” and that “telling women that it’s OK to drink in moderation can be dangerous.” However, the section then ends by stressing that midwives are encouraged to take an individual’s circumstances into account. “We’re not trying to tell people how to live their lives. If someone says ‘I’m going off to a wedding, can I have a glass of champagne?’ that’s different.”

The Brown-Forman website’s advice also suggests that alcohol harms are associated only with “certain drinking patterns”—for example, “Research shows that certain patterns of drinking during pregnancy can be harmful to unborn children.” This misrepresents the association between the amount drunk and the risk of harm. Emphasis on drinking patterns, rather than on actual levels of consumption, is a long-standing alcohol industry strategy (Jernigan, 2012).

#### Framing light drinking, drinking within guidelines, and abstention as equivalent options.

Three alcohol industry–funded bodies appear to frame “light drinking” and abstention as equivalent options for those who are planning to get pregnant or who are pregnant. No examples of this were found among the websites of public health organizations. The Canadian organization Educ’alcool advises that “the safest option is not drinking at all; at the very least, you should cut down on your drinking”—which appears to suggest continuing to drink at some undefined level.

IARD advises that there “is no conclusive evidence of a link between occasional, light, or even moderate drinking during pregnancy and an increased risk for FASD.” “Occasional” and “moderate” drinking, being undefined (or self-defined by the reader), could include a wide range of amounts of alcohol, including drinking at levels harmful to the developing fetus.

#### Confounding: Focusing discussion away from alcohol to other risk factors.

It is well-documented that alcohol, tobacco, and other harmful commodity industries focus on the multifactorial etiology of many health conditions, to distract from the independent harmful effects of those commodities (Petticrew et al., 2018a). Some also appear to do so in relation to pregnancy. For example, Educ’alcool states, “Remember, too, that alcohol is never the only factor involved in the development of the baby. The parents’ basic health, their medical history, their lifestyle, the mother’s diet, external pollutants, tobacco and drug use during pregnancy all have an impact.”

Brown-Forman’s website, which states that it aims to present a “balanced body of research” with “opinions on various sides of issues,” states that the effects of prenatal alcohol exposure on FAS are influenced “by factors including nutrition, metabolism, genetics, and maternal age” and also socioeconomic status.

Similarly, Drinkaware explains how alcohol crosses the placenta, but then it appears to confuse this relationship by stating, “How a baby will be affected depends on how much its mother drinks and the mother’s metabolism. Evidence suggests that diet is also important, with poor maternal nutrition increasing the risk of harm to the unborn baby.”

In these examples it is also unclear what action pregnant women are expected to take about unmodifiable factors such as socioeconomic status, pollutants, and “metabolism.”

Of note, a large proportion of the 12-page IARD document on FAS and FASD focuses on disputing the prevalence, causes, and independent contribution of alcohol to FAS, mixing factual statements with extended discussion of methodological and other uncertainties, and (as above) proposing a number of unmodifiable potential confounders (e.g., being small: “smaller women are more likely to give birth to a child diagnosed with FASD”). The following paragraph is typical:

“. . . there is not necessarily a causal relationship between all potential risk factors and FASD. For example, other maternal risk factors include drinking alone, family members who abuse alcohol, having less stable domestic partnerships, and being at risk for domestic violence. According to a systematic review, FASD births are more common in women with low socioeconomic status and educational level.”

Of note, no examples of these types of argument were found among the websites of public health organizations.

### Quantitative analysis comparing alcohol industry–funded and public health information

In the quantitative comparison between the two types of organizations, alcohol industry–related bodies were statistically significantly less likely to state that no amount of alcohol is safe during pregnancy, to include a statement about risk in the early stages of pregnancy, or to include a biological explanation of how alcohol affects the fetus (although the latter would perhaps not be expected of alcohol industry–funded organizations) (Table 3). The language of alcohol industry–funded bodies was significantly more likely to emphasize uncertainty and less likely (four alcohol industry–funded bodies vs. nine public health bodies) to use the direct imperative (e.g., “stop drinking alcohol if you could be pregnant”). Four alcohol industry–funded bodies, and no public health bodies, used alternate causation arguments (not statistically significant; probably the result of low statistical power).

## Discussion

The Australian alcohol industry–funded body DrinkWise attracted criticism in 2017 for issuing misleading pregnancy posters (*Sydney Morning Herald,* April 17, 2018). The wording, “It’s not known if alcohol is safe to drink when you are pregnant,” was considered misleading, inaccurate, and a mischaracterization of the science. Our findings suggest that this form of misrepresentation of the harms of alcohol during pregnancy may be a wider industry strategy, which includes emphasizing uncertainties and using ambiguous contexts and language to reduce the impact of, or distract from, information on harms (information that may itself be accurate). It may also involve distracting from the independent risks of alcohol consumption by highlighting unmodifiable potential confounders and undermining scientific evidence by emphasizing “balance” and “debate,” and framing scientific evidence as “beliefs.” The same approach has previously been documented in relation to alcohol and cancer risk, and there are many examples from the tobacco industry and other industries across many decades (Table 4) (Conway & Oreskes, 2012, Michaels, 2008).

**Table 4.**
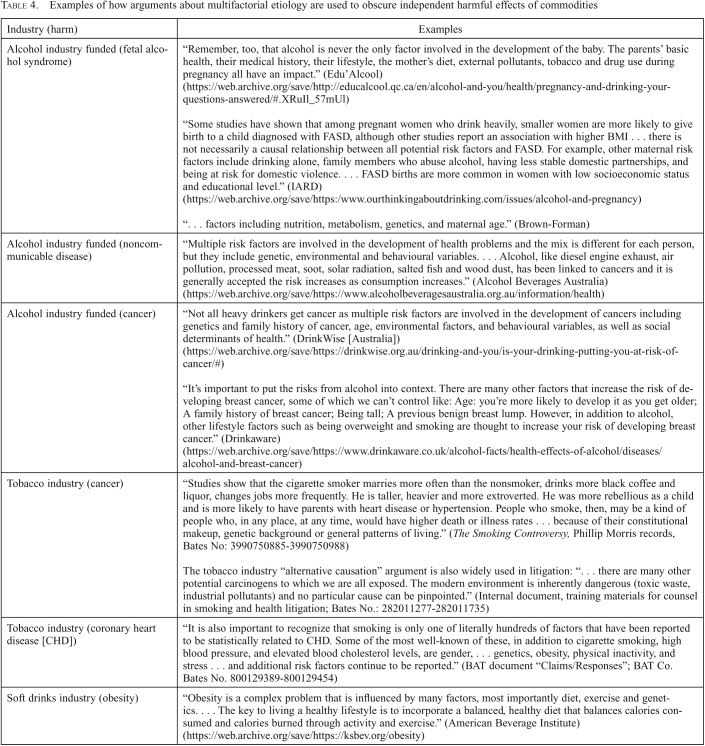
Examples of how arguments about multifactorial etiology are used to obscure independent harmful effects of commodities

Industry (harm)	Examples
Alcohol industry funded (fetal alcohol syndrome)	“Remember, too, that alcohol is never the only factor involved in the development of the baby. The parents’ basic health, their medical history, their lifestyle, the mother’s diet, external pollutants, tobacco and drug use during pregnancy all have an impact.” (Edu’Alcool) (https://web.archive.org/save/http://educalcool.qc.ca/en/alcohol-and-you/health/pregnancy-and-drinking-your-questions-answered/#.XRuIl_57mUl) “Some studies have shown that among pregnant women who drink heavily, smaller women are more likely to give birth to a child diagnosed with FASD, although other studies report an association with higher BMI . . . there is not necessarily a causal relationship between all potential risk factors and FASD. For example, other maternal risk factors include drinking alone, family members who abuse alcohol, having less stable domestic partnerships, and being at risk for domestic violence. . . . FASD births are more common in women with low socioeconomic status and educational level.” (IARD) (https://web.archive.org/save/https:/www.ourthinkingaboutdrinking.com/issues/alcohol-and-pregnancy) “. . . factors including nutrition, metabolism, genetics, and maternal age.” (Brown-Forman)
Alcohol industry funded (noncommunicable disease)	“Multiple risk factors are involved in the development of health problems and the mix is different for each person, but they include genetic, environmental and behavioural variables. . . . Alcohol, like diesel engine exhaust, air pollution, processed meat, soot, solar radiation, salted fish and wood dust, has been linked to cancers and it is generally accepted the risk increases as consumption increases.” (Alcohol Beverages Australia) (https://web.archive.org/save/https://www.alcoholbeveragesaustralia.org.au/information/health)
Alcohol industry funded (cancer)	“Not all heavy drinkers get cancer as multiple risk factors are involved in the development of cancers including genetics and family history of cancer, age, environmental factors, and behavioural variables, as well as social determinants of health.” (DrinkWise [Australia]) (https://web.archive.org/save/https://drinkwise.org.au/drinking-and-you/is-your-drinking-putting-you-at-risk-of-cancer/#) “It’s important to put the risks from alcohol into context. There are many other factors that increase the risk of developing breast cancer, some of which we can’t control like: Age: you’re more likely to develop it as you get older; A family history of breast cancer; Being tall; A previous benign breast lump. However, in addition to alcohol, other lifestyle factors such as being overweight and smoking are thought to increase your risk of developing breast cancer.” (Drinkaware) (https://web.archive.org/save/https://www.drinkaware.co.uk/alcohol-facts/health-effects-of-alcohol/diseases/alcohol-and-breast-cancer)
Tobacco industry (cancer)	“Studies show that the cigarette smoker marries more often than the nonsmoker, drinks more black coffee and liquor, changes jobs more frequently. He is taller, heavier and more extroverted. He was more rebellious as a child and is more likely to have parents with heart disease or hypertension. People who smoke, then, may be a kind of people who, in any place, at any time, would have higher death or illness rates . . . because of their constitutional makeup, genetic background or general patterns of living.” (*The Smoking Controversy,* Phillip Morris records, Bates No: 3990750885-3990750988) The tobacco industry “alternative causation” argument is also widely used in litigation: “. . . there are many other potential carcinogens to which we are all exposed. The modern environment is inherently dangerous (toxic waste, industrial pollutants) and no particular cause can be pinpointed.” (Internal document, training materials for counsel in smoking and health litigation; Bates No.: 282011277-282011735)
Tobacco industry (coronary heart disease [CHD])	“It is also important to recognize that smoking is only one of literally hundreds of factors that have been reported to be statistically related to CHD. Some of the most well-known of these, in addition to cigarette smoking, high blood pressure, and elevated blood cholesterol levels, are gender, . . . genetics, obesity, physical inactivity, and stress . . . and additional risk factors continue to be reported.” (BAT document “Claims/Responses”; BAT Co. Bates No. 800129389-800129454)
Soft drinks industry (obesity)	“Obesity is a complex problem that is influenced by many factors, most importantly diet, exercise and genetics. . . . The key to living a healthy lifestyle is to incorporate a balanced, healthy diet that balances calories consumed and calories burned through activity and exercise.” (American Beverage Institute) (https://web.archive.org/save/https://ksbev.org/obesity)

These findings are consistent with the tactics identified by Savell et al. (2016), who explain how alcohol industry bodies “shift the focus away from the harm of their own products to emphasize instead individual responsibility of the consumer” (p. 26). The use of the term *responsible drinking* by the alcohol industry has been shown to be strategically ambiguous, as is the emphasis found in alcohol industry documents on unquantified “drinking patterns.” These ambiguities avoid any commitment to specific consumption levels that might harm business (Jernigan, 2012; Petticrew et al., 2018a, 2018b; Smith et al., 2006). Our findings show that some alcohol industry–funded bodies also do this by framing “light drinking” as an equivalent to abstention.

The emphasis on undefined “heavy” drinking on some of these alcohol industry–funded websites may also be ambiguous. When terms such as *heavy* are not translated into units of alcohol, people may be unlikely to classify themselves as “abusing alcohol” even if they regularly drink above guideline levels, particularly as people underreport their drinking (Boniface et al., 2014). Negative terms such as alcohol *abuse* may also actively discourage readers from associating themselves with this description.

The direct and unambiguous language used by public health bodies is also found to be different from the often-ambiguous language used by alcohol industry–funded bodies. Furthermore, the positioning of information on pregnancy on alcohol industry websites, such as the Drinkaware website, may also make it less visible, or may de-emphasize its importance by placing it below alcohol trivia.

We noted that on some websites the positioning of the information on the webpage appears to dilute its importance and/or present mixed messages. For example, on the Drinkaware website, the sections on pregnancy appear on the webpage titled “Health effects of alcohol.” The page has 45 sections, of which the last four are sections on pregnancy, breastfeeding, FAS, and fertility, requiring the user to scroll down approximately nine pages to access the information (see: https://web.archive.org/save/https://www.drinkaware.co.uk/alcohol-facts/health-effects-of-alcohol). This positioning is well below, for example, the sections on “How does alcohol affect my beer belly?” and “Why does alcohol make you pee more?” Similarly, on Pernod Ricard’s “Wise Drinking” app, a statement that FAS is a “pattern of physical and mental defects that can develop in a fetus in association with high levels of alcohol consumption during pregnancy” is found alongside trivia such as “the term Champagne can only be used for wines produced in the Champagne region.” Other examples suggest that further analysis of the positioning of information on these websites would be important.

Across alcohol industry–funded organizations, there appears to be a consistent approach to the delivery of information on alcohol consumption and pregnancy: This involves in many cases avoiding the unambiguous emphasis on abstention and instead using language that encourages the maintenance of some level of alcohol consumption during pregnancy. Even when information is included (e.g., about risk throughout pregnancy), it may be simultaneously undermined by its framing, language, and/or positioning. One possible reason is that women are a crucial part of the alcohol market, as has been pointed out in relation to alcohol consumption and breast cancer risk (Connor, 2017). Pregnancy, therefore, may represent a significant commercial threat, if it means that women’s long-term drinking patterns change as a result of initially drinking less while planning a pregnancy and then during the 9 months of pregnancy—for example, if the initial reduction leads to longer term reductions in their drinking (Urban et al., 2016). This could represent a significant loss to the alcohol market, with the added risk that in such women abstention may become normalized. The strategies outlined in this study (omission, framing, and linguistic ambiguities) therefore may reflect the alcohol industry’s protection of the female market, with the common goal of “nudging” women toward continuing to consume alcohol during pregnancy.

Other strategies may include providing inadequate or no pregnancy warning information on product labels, as well as opposing attempts by governments or health authorities to introduce health information for consumers (Farrell-Low, 2018; Petticrew et al., 2016). These should also be seen as part of a wider set of industry tactics that includes manipulating the evidence base, lobbying, and constituency building (forming alliances with other sectors, organizations, or the public to give the impression of larger support for the industry’s position) (Savell et al., 2016). More generally, the alcohol industry involves itself in providing health information because it can then can portray itself as “part of the solution” and therefore play a greater role in the regulatory landscape. This echoes strategies adopted by the tobacco industry when it was faced with the growing, unequivocal evidence of the harms of smoking. On the advice of one of America’s leading public relations firms, Hill and Knowlton, the tobacco industry sought to capture and control the science and its dissemination rather than ignore or deny it, presenting itself as a great supporter of science (Brandt, 2012).

The wording on the IARD website is of particular interest, as it seems to place significant emphasis on women in “less stable domestic partnerships” and “women with low socioeconomic status and educational level.” It is conceivable that this may be intended to make the message appear less relevant to women of higher socioeconomic status, among whom FASD is less likely to be diagnosed and who are at the same time, because of their income, a valuable part of the alcohol market. However, this would require further analysis.

Finally, we emphasize that there are indeed uncertainties and complexities in the area of alcohol and health, not the least in defining the benefits of risks and harms from “light” drinking. However, we have shown that the information from alcohol industry–funded organizations systematically differs from that disseminated by public health bodies, in ways that likely benefit or protect the female alcohol market. In addition, the alcohol industry’s competing and ambiguous public narratives about the harms of alcohol consumption may fail to reinforce, or may directly undermine, government advice.

### Strengths and limitations

Comparative analysis of messaging from alcohol industry–funded and public health organizations is uncommon and is a strength of this study. In particular, analysis of linguistic characteristics allows for new insights, and such analyses of industry discourses would be extremely valuable.

A key limitation is that only websites in English were included. Also, alcohol industry–funded bodies disseminate information in other media, and these should be analyzed. A further limitation is that the quantitative analysis has low statistical power, given the relatively small number of websites. Note also that we do not have direct evidence, such as written or oral statements, of industry intent in relation to the above strategies; and that website content may have changed since completion of this analysis in May 2019. In addition, not all these organizations may be wholly industry funded; some may have other non-industry funding.

### Conclusion

The alcohol industry involves itself in providing health information so it can portray itself as “part of the solution” and, therefore, play a greater role in the regulatory landscape. Such initiatives have repeatedly been shown to be ineffective and potentially harmful. Our findings suggest that alcohol industry corporate social responsibility bodies may use strategic ambiguity and other informational tactics to “nudge” women toward continued drinking in pregnancy to protect the female alcohol market.

This study provides further evidence that alcohol industry corporate social responsibility organizations pose a potential risk to public health, specifically to the health of pregnant women and unborn children, and should have no role in disseminating health information. The public should now be made aware of the risks in using these sources.

## Conflict of Interest Statement

The authors have no conflict of interest to declare.

## Acknowledgment

We thank Ben Hawkins for his advice on methods, interpretation of the findings, and helpful comments on earlier drafts of the article. We also thank the reviewers, whose detailed comments helped strengthen the analysis. The bulk of the research was conducted at London School of Hygiene and Tropical Medicine as part of Audrey W. Y. Lim’s master’s thesis. Ethical approval for this study was not required, as it entailed a secondary analysis of publicly available data and documents.
